# Postoperative delirium in geriatric patients with hip fractures

**DOI:** 10.3389/fnagi.2022.1068278

**Published:** 2022-12-22

**Authors:** Yang Chen, Shuai Liang, Huiwen Wu, Shihao Deng, Fangyuan Wang, Ciren Lunzhu, Jun Li

**Affiliations:** ^1^Department of Orthopedics, The Second Hospital of Anhui Medical University, Hefei, China; ^2^Institute of Orthopedics, Research Center for Translational Medicine, The Second Hospital of Anhui Medical University, Hefei, China; ^3^Department of Orthopedics, Shannan City People’s Hospital, Shannan, China

**Keywords:** postoperative delirium, geriatric patients, hip fractures, orthogeriatrics, prevention, treatment

## Abstract

Postoperative delirium (POD) is a frequent complication in geriatric patients with hip fractures, which is linked to poorer functional recovery, longer hospital stays, and higher short-and long-term mortality. Patients with increased age, preoperative cognitive impairment, comorbidities, perioperative polypharmacy, and delayed surgery are more prone to develop POD after hip fracture surgery. In this narrative review, we outlined the latest findings on postoperative delirium in geriatric patients with hip fractures, focusing on its pathophysiology, diagnosis, prevention, and treatment. Perioperative risk prediction, avoidance of certain medications, and orthogeriatric comprehensive care are all examples of effective interventions. Choices of anesthesia technique may not be associated with a significant difference in the incidence of postoperative delirium in geriatric patients with hip fractures. There are few pharmaceutical measures available for POD treatment. Dexmedetomidine and multimodal analgesia may be effective for managing postoperative delirium, and adverse complications should be considered when using antipsychotics. In conclusion, perioperative risk intervention based on orthogeriatric comprehensive care is the most effective strategy for preventing postoperative delirium in geriatric patients with hip fractures.

## Introduction

Osteoporotic hip fracture is an important public health concern, with a reported one-year mortality of 12.7% in geriatric patients (Aharonoff et al., 1997). It affects women more frequently than males (Brauer et al., 2009), most likely because post-menopausal women have a higher incidence of osteoporosis (Cummings and Melton, 2002). Hip fracture in geriatric patients is linked to poor functional prognosis and a high frequency of postoperative complications, such as postoperative delirium (POD), pneumonia, urinary tract infections, deep vein thrombosis, and bleeding (Fischer et al., 2021; Schrøder et al., 2022). A third of geriatric patients encounter one or more postoperative complications, and 7.2% of patients experience multiple complications (Merchant et al., 2005). It is reported that compared with senior patients without fractures, those with hip fractures had a higher rate of postoperative mental problems (Kuo et al., 2021).

Postoperative delirium (POD) is one of the most common complications in geriatric patients with hip fractures (Merchant et al., 2005). It is a postoperative cognitive disturbance defined by abrupt and variable impairment in attention and awareness, with reported incidences varying from 4.7 to 74% (Maldonado, 2017; Flikweert et al., 2018; Connolly et al., 2020). POD usually occurs between postoperative days 2–5 (Jin et al., 2020). When patients develop POD, it is usually the most distressing component of the perioperative experience, adding to their personal, medical, and financial burdens (Leslie et al., 2008). POD is associated with increased hospital length of stay (Hecht et al., 2019), poor rehabilitation outcomes, such as impaired functional and cognitive recovery, even the onset of new dementia (Low et al., 2021; Meyer et al., 2021; Pereira et al., 2021), worsened mobility (Ouellet et al., 2019), and a higher risk of both short-term and long-term mortality (Bai et al., 2020; Liu et al., 2021). Additionally, postoperative delirium is a strong indicator for nursing home admission (de Jong et al., 2019). In light of these poor consequences and the high prevalence of POD in geriatric patients with hip fractures, early identification of patients at risk as well as implementation of prophylactic measures to reduce the frequency of POD is extremely desirable (Smith et al., 2017). The underlying pathophysiology of postoperative delirium in geriatric patients with hip fractures is still unclear, most studies focus on the involvement of neuroinflammation, neurotransmitters, and metabolism abnormality. Currently, there are still few effective measures for POD treatment. The most effective approach to preventing POD is to minimize its risk factors, and several risk prediction tools have been developed for risk stratification in geriatric patients with hip fractures. Additionally, avoidance of perioperative benzodiazepine administration and comprehensive multidisciplinary care play a significant role in POD management.

This review aims to describe current research advances on postoperative delirium in geriatric patients with hip fractures and to provide effective and feasible strategies for the prevention and treatment of POD.

## Epidemiology and risk factors

Despite that the prevalence of POD in the general surgical population is only 2–3%, it can occur in up to 50–70% of high-risk patient groups (Watne et al., 2016). In particular, hip fracture surgery is associated with up to 70–80% risk of postoperative delirium. There are several reasons for this: a hip fracture is frequently seen in elderly patients who are frail; perioperative pain is a major concern; and the surgery is typically performed in an emergency setting with little chance for preoperative optimization (Jin et al., 2020).

The perioperative risk factors for POD in geriatric patients with hip fractures can be predisposing and precipitating. As is shown in the literature (Smith et al., 2017; Swarbrick and Partridge, 2022; Wittmann et al., 2022), predisposing risk factors include increased age, sensorial deficits, prior cognitive impairment, multimorbidity, malnutrition, and frailty, as well as precipitating risk factors including surgical procedures, ventilation time, and intensive care stay. In addition, there are also several independent predictors for POD in geriatric patients with hip fractures (Koskderelioglu et al., 2017; Mosk et al., 2017; Smith et al., 2017; Yang et al., 2017; Wang et al., 2018; Chen et al., 2020; Tantardini et al., 2020; Xing et al., 2020; Zhou et al., 2021; Table 1).

**Table 1:** Risk factors and predictors for postoperative delirium in geriatric patients with hip fractures.

Preoperative risk factors and predictors	Advanced age (> 75 years or 71.5 years)
	Male
	ASA classification (> 2 level)
	Institutionalization
	Cognitive impairment or dementia
	Low mini-mental state examination score
	Multiple comorbidities (e.g., heart failure, diabetes, stoke, depression)
	Hyponatremia or hypernatremia
	Acetylcholine <7.75 mmol/l or choline acetyltransferase <30.15 pg./ml
	Low hemoglobin levels
	Low prognostic nutritional index level
Intraoperative risk factors and predictors	Total hip arthroplasty
	Intraoperative bleeding
	High amount of blood transfusion
Postoperative risk factors and predictors	Pneumonia or a urinary tract infection
	Pain
	Morphine usage
	Postoperative acute kidney injury

ASA, American Society of Anesthesiologists.

Increased age is an independent risk factor associated with POD in geriatric patients with hip fractures. Patients older than 71.5 years of age with hip fractures have a more than threefold increased risk of developing postoperative delirium (Xing et al., 2020), and the hypoactive subtype is more likely to develop in people over the age of 80 (Stagno et al., 2004). This may be because the risk of endothelial dysfunction and atherosclerosis increases with age, putting elderly patients at increased risk of cerebral embolism (Rudolph et al., 2009). Additionally, the age-related loss of cholinergic reserve is also a significant contributor. Interestingly, males are more prone to develop delirium after hip fracture surgery (Yang et al., 2017; Haynes et al., 2021), despite women having a larger frequency of hip fractures (Edelstein et al., 2004). A 20–45% risk of postoperative delirium is linked to emergency surgery, which is 1.5–3 times higher than the risk for comparable elective surgery (Ansaloni et al., 2010; Chaiwat et al., 2019). Surgery type may have an influence on POD in geriatric patients with hip fractures. Patients who underwent total hip arthroplasty were more likely to develop POD (OR 2.21; 95% CI 1.16–4.22; Bruce et al., 2007; Yang et al., 2017). The location of the fracture (transcervical fracture, intertrochanteric fracture, and subtrochanteric fracture) seems has no association with the occurrence of POD, according to existing literature (Smith et al., 2017; Xing et al., 2020). Preoperative dementia is also an independent risk factor for POD in elderly patients with hip fractures. Moreover, postoperative delirium plays moderation and mediation effects between preoperative dementia and postoperative adverse events. The effects of preoperative dementia on mortality, readmission, and revision surgery were dramatically enhanced by postoperative delirium (Haynes et al., 2021). ASA physical status classes 2 and 3 and the number of medical comorbidities were significant variables in assessing the risk of POD. The modified Charlson’s Comorbidity Index (CCI) is a commonly used tool for assessment of medical comorbidities (Charlson et al., 1987). It is reported that 94% of hip fracturs patients with POD were associated with at least a medical complication (Mullen and Mullen, 1992). Postoperative infection, such as urinary tract infection and pneumonia, can also increase the POD risk in hip fracture patient. It may cause significant delays in cognitive processing speed, especially when it comes to memory retrieval (Bucks et al., 2008). Additionally, the risk of developing POD is increased by impaired vision or hearing because the elderly person cannot properly perceive the stimuli in the hospital environment (Staus, 2011).

## Pathophysiological changes

The pathophysiology of postoperative delirium in elderly patients with hip fractures has not been entirely explored yet, and widely accepted hypotheses focus on the involvement of neuroinflammation, neurotransmitters, and metabolic abnormality.

### Neuroinflammation

**Figure 1:**
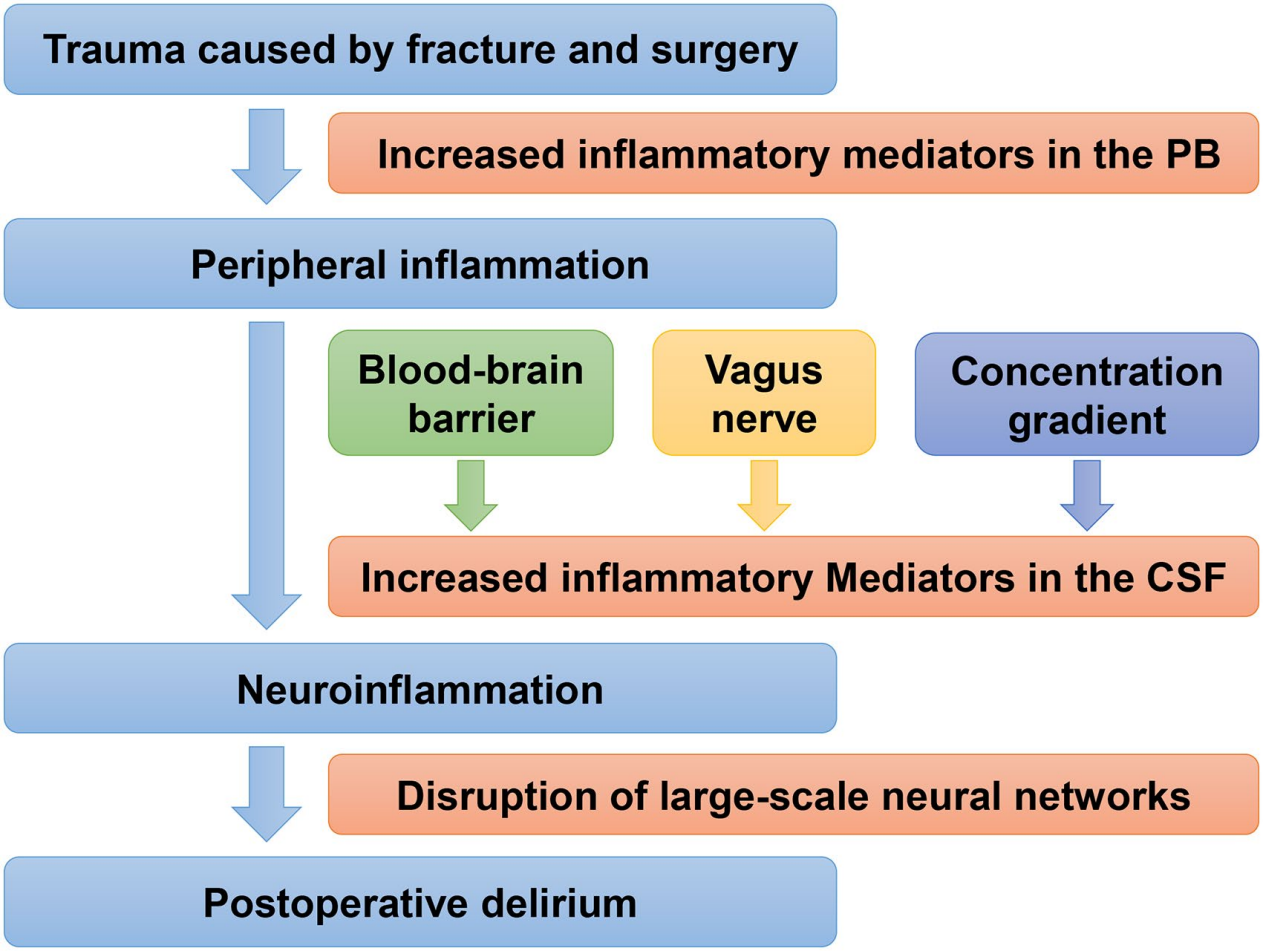
Neuroinflammation mechanism of postoperative delirium in geriatric patients with hip fractures. PB, peripheral blood; CSF, cerebrospinal fluid. Trauma caused by fracture and surgery is associated with increased systemic inflammatory mediators, which are transported from the periphery to the brain *via* three primary pathways, resulting in neuroinflammation, and finally lead to POD.

One generally accepted pathophysiological explanation for postoperative delirium in elderly patients with hip fractures is neuroinflammation (Figure 1). Surgical injuries act on Toll-like receptors through damage-associated molecular patterns or pathogen-associated molecular patterns to initiate an intracellular inflammatory response, and inflammatory mediators such as TNF - α are released into the brain, causing aseptic inflammation in the CNS, resulting in POD (Alam et al., 2018). Previous studies have shown that trauma caused by fracture and surgery is associated with increased perioperative systemic inflammatory mediators (Cape et al., 2014; Neerland et al., 2016). These peripheral inflammatory mediators are transported to the brain *via* three primary pathways: (a) Peripheral inflammation causes the loss of structural and functional integrity of the blood–brain barrier. Thus, inflammatory factors can cross the blood–brain barrier and enter the brain through capillary epithelial cells (Yang et al., 2017); (b) There are transporters in the afferent nerves of the vagus, which can directly transport the peripheral inflammatory substances to the corresponding nerve nuclei; (c) The increased concentration of inflammatory factors in plasma makes it possible for the inflammatory mediators to diffuse to brain tissue through the concentration gradient. The accumulation of central inflammatory mediators leads to the disruption of the large-scale neural networks in the brain, such as suppression of hippocampal plasticity and neurogenesis (Ekdahl et al., 2003; Prieto et al., 2019), neurotoxicity, and neuronal apoptosis (Tian et al., 2018). These damages ultimately result in the occurrence and progress of POD.

### Neurotransmitters

The alteration in neurotransmitters is also a possible mechanism for the occurrence and development of POD, among which the most prominent is the deficiency or dysfunction of the cholinergic system (Adam et al., 2020). A prospective observational study demonstrated that the level of acetylcholine in the cerebral spinal fluid was significantly decreased in geriatric patients with postoperative cognitive dysfunction (Chen et al., 2020). Moreover, the cholinergic system interacts significantly with other neurotransmitter systems, and POD is also correlated with the malfunction of these other neurotransmitter systems. Particularly, acetylcholine deficiency is accompanied by an excess of dopamine, which is linked to critical postoperative delirium (Trzepacz, 2000). However, a randomized controlled trial of 261 patients found that postoperative administration of physostigmine did not affect the prevention of POD after liver resection (Spies et al., 2021), which may indicate the underlying mechanisms leading to neurotransmitter alterations still need to be explored to find effective drug targets.

### Neurovascular changes

Postoperative delirium is more likely to occur in elderly patients with neurovascular changes. A review explored the association of neurodegenerative and neurovascular changes with the occurrence of POD (Kant et al., 2017), and it found that neurovascular changes, such as white-matter hyperintensities (Hatano et al., 2013; Root et al., 2013), preoperative old cerebral infarct (Otomo et al., 2013; Maekawa et al., 2014) and markers of white matter integrity (Shioiri et al., 2010; Cavallari et al., 2016), appear to be consistently associated with the occurrence of POD. Furthermore, these changes may even help to identify patients at increased risk of POD. Kyeong et al. (2018) developed a prediction model for estimating the probability of POD in geriatric patients with femoral neck fractures, with a correct classification rate of 86%. This model consists of three neural predisposing factors: the neuroticism score, the amplitude of low-frequency fluctuation in the dorsolateral prefrontal cortex, and the density of gray matter in the caudate or suprachiasmatic nucleus. It may accurately predict the likelihood of postoperative delirium by reflecting the fundamental pathophysiology.

### Metabolic abnormality

Metabolic abnormality is shown to be associated with the occurrence and progression of POD. In a metabonomic study of geriatric hip fracture patients, it was discovered that, while glycolysis products were higher in POD patients after surgery, levels of ω3 and ω6 fatty acids were lower in those both before and after surgery, as well as intermediate levels of the tricarboxylic cycle and the branched-chain amino acid/aromatic amino acid ratio (Guo et al., 2019). These metabolic abnormalities most likely reduce the ability of the brain to provide neuroprotection and take part in neuroinflammatory responses. Another study applied untargeted metabolomics techniques to investigate the change of serum metabolites in POD patients undergoing cardiopulmonary bypass, and found that low serum lipid metabolic phosphatidylinositol was linked to an increased risk of POD in the geriatric patient, which may shed fresh light on the pathophysiology of POD (Huang et al., 2022).

### Serum biomarkers and predictors

High clinical significance can result from the identification of predictive biomarkers that allow for early risk stratification of hip fracture patients who are vulnerable to POD. The incidence and severity of POD in hip fracture patients were associated with higher levels of white blood cells, neutrophil/lymphocyte ratio, neutrophil percentage, and lower levels of mean platelet volume and basophil percentage (Thisayakorn et al., 2021; Li et al., 2022), which further demonstrated that immune-inflammatory processes are involved in the pathophysiology of POD. Prognostic Nutritional Index (PNI) is a convenient and accurate way to quantify nutritional status, and it was calculated as 10 × albumins (g/dl) + 0.005 × total lymphocyte count (per mm^3^; Onodera et al., 1984). It is reported that lower preoperative PNI value (cut-off value: 47.45, sensitivity: 86.0%, specificity: 51.9%, OR: 2.88, 95% CI: 1.25–6.64, *p* = 0.012) was associated with increased POD risk in geriatric patients with hip fractures, and it was a predictor for POD with an area under the curve of 0.686 (95% CI: 0.604–0.767, *p* < 0.001; Xing et al., 2020). The neuropeptide galanin is a neuromodulator ubiquitously present in the central and peripheral nervous systems as well as nonneural tissues, and its levels are abnormally high in depressive and dementia-like diseases (Counts et al., 2001; Alexandris et al., 2015). It is reported that the increased serum neuropeptide galanin level can predict postoperative cognitive dysfunction in geriatric patients with hip fractures (*p* = 0.035). However, the association between serum neuropeptide galanin level and POD in geriatric patients with hip fractures has not been investigated yet.

## Diagnosis

In the current, the diagnosis of postoperative delirium in geriatric patients with hip fractures mainly relies on clinical characteristics. Patients with POD frequently exhibit either hyperactive or hypoactive types, or a mixed type, which alternates between these motor subtypes (Vlisides and Avidan, 2019). The hypoactive type is more prevalent in elderly people, frequently goes undiagnosed, and is linked to higher rates of other postoperative complications and mortality (Oh et al., 2017). Additionally, in the clinical setting, the workload of clinicians is so high that it is difficult to detect POD in time, so the underdiagnosis rate of delirium remains high. Studies found a delirium underdiagnosis rate of 60% in elderly patients with cancer and an even higher rate of 84.6% in elderly patients in the emergency setting (de la Cruz et al., 2015; Boucher et al., 2019).

### Diagnosis criteria

Delirium has been defined as a disturbance in attention and consciousness based on the following standards in the Diagnostic and Statistical Manual of Mental Disorders, 5th Edition (DSM-5; American Psychiatric Association, 2013; European Delirium Association, American Delirium Society, 2014): (A) Disturbance in attention (i.e., reduced ability to focus, sustain, or shift attention) and awareness (reduced orientation to the environment); (B) The disturbance develops over a short period (usually hours to a few days), represents an acute change from baseline attention and awareness, and tends to fluctuate in severity during the course of the day; (C) An additional disturbance in cognition (e.g., memory deficit, disorientation, language, visuospatial ability, or perception); (D) The disturbances in Criteria A and C are not better explained by a pre-existing, established, or evolving neurocognitive disorder and do not occur in the context of a severely reduced level of arousal such as a coma; (E) There is evidence from the history, physical examination, or laboratory findings that the disturbance is a direct physiological consequence of another medical condition, substance intoxication or withdrawal (i.e., due to a drug of abuse or to a medication), or exposure to a toxin, or is due to multiple etiologies.

### Diagnosis instrument

Prompt diagnosis of delirium in geriatric patients with hip fractures is the first key step in its appropriate treatment, and the diagnosis approaches in these patients must be convenient and easy to operate. The widely used instrument for POD diagnosis in the clinical setting is the confusion assessment method (CAM) developed in 1990, which is a bedside structured interview with the patient (Inouye et al., 1990). It concentrates on the most notable clinical characteristics of delirium: (1) acute change in mental status with a fluctuating course; (2) inattention; (3) disorganized thinking; and (4) altered level of consciousness. To make the diagnosis of delirium, (1) and (2) must be present, and the patient must have signs of either (3) or (4). Additionally, in the intensive care unit (ICU) setting, POD is often diagnosed using CAM-ICU, a 2-min version of CAM that is convenient to use and highly accurate (Ely et al., 2001; Heo et al., 2011).

The Mini-mental state examination (MMSE) is also a wildly used instrument for the diagnosis of POD. It is intended to assess abilities related to orientation, memory, attention, object identification, compliance with verbal and written instructions, free writing of sentences, and copying complicated polygons (Folstein et al., 1975). The Organic brain syndrome (OBS) scale mainly assesses two dimensions: disorientation and confusion. A disorientation subscale based on an interview describes the patient’s capacity for time, place, and self-identity orientation; a confusion subscale based on the researcher’s or the nursing staff’s observations that assess a variety of cognitive, perceptual, emotional, and personality alterations, as well as physical and practical impairments, and variations in the clinical condition (Berggren et al., 1987).

There is a study that compared 11 instruments for delirium assessment, and it found that the CAM is the best bedside delirium assessment tool, which takes 5 min to administer, while the MMSE (score < 24) is the least useful for identifying a patient with delirium (Wong et al., 2010). Furthermore, Marcantonio et al. (2014) have developed a 3-min diagnostic interview based on the CAM, called 3D-CAM, which assesses 4 CAM features with cognitive tests, patients question, and interviewer observation. It was demonstrated to have good sensitivity [95% (84, 99%)] and specificity [94% (90, 97%)] in comparison to a reference standard, and was shown to be effective in POD diagnosis in geriatric patients with hip fractures (Neuman et al., 2021).

### Severity assessment

Increasing evidence indicates that delirium, as well as its severity, is associated with worse outcomes (de Miguel et al., 2018; Sieber et al., 2018). It is vital to measure delirium severity to assess prognosis and to monitor treatment response. The most frequently used instruments to rate the severity of POD include the Confusion Assessment Method-Severity (CAM-S; Inouye et al., 2014), the Delirium Rating Scale-Revised-98 (DRS-R-98; de Rooij et al., 2006), and the Memorial Delirium Assessment Scale (MDAS) (Breitbart et al., 1997). The same fundamental concept of delirium severity is measured by each of these delirium instruments, with a high degree of correlation (Gross et al., 2018).

However, postoperative delirium is characterized by acute onset and recurrent fluctuations. It is difficult for the bedside assessment instruments such as the CAM-S to dynamically grasp the recurrent fluctuations, and the accuracy of these scales in assessing POD depends on the training level of assessors. Furthermore, it is demonstrated that the electrocardiogram (ECG) slowing, such as a composite of generalized theta or delta slowing (OR 10.3, 95% CI, and 5.3–20.1), is associated with delirium severity and poor clinical outcomes (Kimchi et al., 2019). Recently, an automated physiologic process that quantifies the presence and severity of delirium directly has been reported, called the Electroencephalographic Confusion Assessment Method Severity Score (E-CAM-S; van Sleuwen et al., 2022). It is based on a learning-to-rank machine learning model of forehead electroencephalography signals, with a level of performance comparable to conventional interview-based clinical assessment. Tesh et al. (2022) developed the Visual EEG Confusion Assessment Method Severity (VE-CAM-S) for the diagnosis of delirium severity, which was strongly associated with clinically important outcomes. Those ECG-based assessment methods may help close the delirium diagnostic gap and develop more effective delirium treatments and prevention strategies.

## Prevention and treatment

Hitherto, there is still no effective strategy to treat POD in geriatric patients with hip fractures, and studies on the management of postoperative delirium mainly concentrate on its precautions. According to a 2020 meta-analysis, 91% of trials about POD focused on prevention and only 9% on its treatment (Pieri et al., 2020). The prevention and treatment measures of POD in geriatric patients with hip fractures can be divided into non-pharmacological and pharmacological interventions.

### Non-pharmacological interventions

#### Risk prediction and reduction

Early identification of hip fracture patients with POD risk and adoption of effective intervention are important to prevent the occurrence and development of POD. The risk factors and predictors of POD in geriatric patients with hip fractures are listed in Table 1. Clinicians should be vigilant for POD in patients who present with these features during the perioperative period. In recent years, a variety of risk prediction models have been developed to predict POD risk in geriatric patients with hip fractures (Kim et al., 2020; Oberai et al., 2021; Shen et al., 2022; Table 2), which enable delirium risk stratification for hip fracture patients and facilitate the development of strategies for POD. Adapted from the AWOL (Age, WORLD backward, Orientation, illness severity) delirium prediction tool for medical inpatients Douglas et al. (2013), Whitlock et al. (2020) developed a perioperative delirium risk stratification tool called AWOL-S (Age, WORLD backward, Orientation, illness severity, Surgery-specific risk) for elective surgical patients. The AWOL-S tool shows a moderate level of accuracy for delirium prediction in patients undergoing elective surgery, with 75% sensitivity and 59% specificity. According to its procedure-specific delirium risk score, hip fracture surgery belongs to the high-risk level, which needs continuing delirium prevention interventions after discharge from anesthesia care. Bishara et al. (2022) developed a delirium risk prediction model based on machine learning, which demonstrated excellent calibration compared with models developed with traditional logistic regression. It is not limited by time and space and can be repeated and standardized to assess patients. However, the incidence of POD in this study (5.3%) was lower than commonly reported POD rates, which may be owing to the inclusion of a younger population and all procedures, even those considered to have a low risk of delirium, such as gynecologic, urologic and plastics. Future studies could validate the sensitivity and specificity of this model specifically in elderly patients with hip fractures.

**Table 2:** Risk prediction models for postoperative delirium in geriatric patients with hip fractures.

Source	Variables	OR (95% CI)	Scores	AUC (95% CI)
Kim et al. (2020)	Preoperative delirium	8.32 (6.78–10.21)	8	Derivation cohorts: 0.77 (0.76–0.78); Validation cohorts: 0.77 (0.75–0.79)
	Preoperative dementia	2.38 (2.05–2.76)	3	
	Age			
	70–79 year	1.60 (1.20–2.12)	2	
	80–89 year	2.09 (1.59–2.74)	2	
	≥ 90 year	2.43 (1.82–3.23)	3	
	Medical co-management	1.43 (1.13–1.81)	1	
	ASA physical status III - V	1.40 (1.14–1.73)	1	
	Functional dependence	1.37 (1.17–1.61)	1	
	Smoking	1.36 (1.07–1.72)	1	
	SIRS/sepsis/septic shock	1.34 (1.09–1.65)	1	
	Preoperative use of mobility aid	1.32 (1.14–1.52)	1	
Oberai et al. (2021)	Age > 80 year	1.6 (1.4–1.9)	5	Derivation cohorts: 0.742; Validation cohorts: 0.746
	Male gender	1.3 (1.1–1.5)	2	
	Mobilization opportunity	1.9 (1.4–2.6)	6	
	Surgery delay	1.7 (1.2–2.5)	6	
	Cognition not assessed	1.5 (1.3–1.9)	4	
	Cognition impaired	1.7 (1.3–2.1)	5	
	Prior impaired cognition or known dementia	3.90 (3.2–4.7)	14	
Shen et al. (2022)	Preoperative delirium	4.21 (3.25–9.14)	4	Derivation cohorts: 0.848 (0.72–0.90); Validation cohorts: 0.833 (0.68–0.89)
	CVA with the modified Rankin scale			
	≥ 4	3.17 (1.16–5.06)	3	
	2–3	2.25 (1.26–4.29)	2	
	Diabetes with random glucose level			
	> 13 mmol/l	2.43 (1.32–2.99)	2	
	8–13 mmol/l	1.36 (1.15–1.67)	1	
	CCI score			
	≥ 9	2.32 (1.69–4.83)	2	
	6–8	1.29 (1.03–2.52)	1	
	Age			
	≥ 80 year	1.87 (1.25–2.50)	2	
	70–79 year	1.11 (1.07–2.19)	1	
	Application of Benzodiazepines in surgery	1.44 (1.24–3.17)	1	
	Surgical delay ≥ 2 days	1.15 (1.13–1.28)	1	
	Creatine ≥ 90 μmol/l	1.09 (1.02–1.13)	1	
	Active smoker	1.05 (1.04–1.94)	1	

OR, odds ratio; CI, confidence interval; AUC, area under the curve; ASA, American Society of Anaesthesiologists; SIRS, systemic inflammatory response syndrome; CVA, cerebrovascular accident; CCI, Carlson comorbidity index.

#### Orthogeriatric comprehensive care

**Figure 2:**
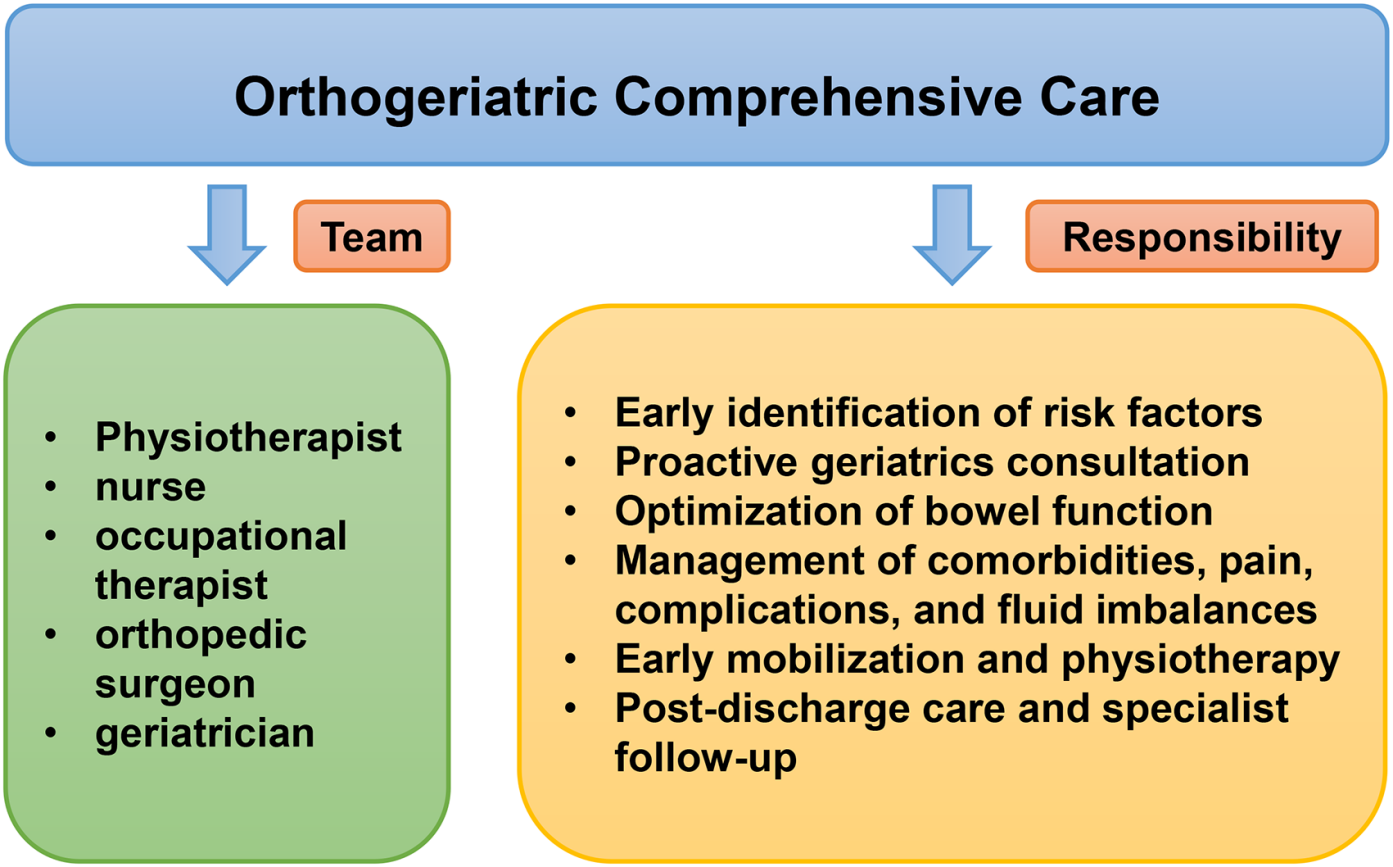
Orthogeriatric comprehensive care in geriatric patients with hip fractures for management of postoperative complication.

Orthogeriatric comprehensive care is a multidisciplinary methodology of systematic assessment and optimization for elderly patients following orthopedic surgery, which plays a significant role in the progress of geriatric patients with hip fractures (Li et al., 2021). The orthogeriatric comprehensive care team usually includes orthopedic surgeons, geriatricians, physiotherapists, occupational therapists, and nurses. The primary responsibility of the team includes the identification of risk factors, optimization of nutrition and bowel function, as well as management of comorbidities, complications, pain, and fluid imbalances for geriatric patients with hip fractures (Figure 2). A 2020 meta-analysis showed that comprehensive interventions, such as education, awareness, and multidisciplinary collaboration around POD prevention and management, significantly reduced the prevalence of POD (Igwe et al., 2020).

A substantially reduced incidence of POD in patients with hip fractures was shown to be associated with proactive geriatrics consultation and orthogeriatric co-management during the perioperative period (Marcantonio et al., 2001; Pollmann et al., 2021). In a meta-analysis of 1840 elderly patients with hip fractures (Wang et al., 2017), the incidence of POD in the comprehensive geriatric care group was significantly reduced compared to the routine orthopedic care group (OR 0.71; 95% CI, 0.57–0.89; *p* = 0.003). It also revealed that comprehensive geriatric care may accelerate cognitive function recovery after hip fracture surgery. However, these findings should be interpreted cautiously in light of the limitations of this study. For example, the instruments used to diagnose patients with POD are inconsistent, with variable sensitivity and specificity, and the influencing factors such as staff member experience, anesthetic method, and inclusion/exclusion criteria would differ between studies.

Additionally, Enomoto et al. (2021) created a clinical program called the Delirium Team Approach, which included educational sessions, reviews of preprinted doctor orders, and routine delirium screening. They found that implementation of this program could shorten postoperative hospital stays and reduce the incidence of POD in elderly patients after cardiovascular surgery. Furthermore, more multicenter RCTs with excellent methodology are required to gather proof of the effect of comprehensive orthogeriatric care on POD prevention in geriatric patients with hip fractures.

#### Choice of anesthetic technique

The choice of regional anesthesia or general anesthesia was thought to have an impact on the occurrence of POD previously (Ravi et al., 2019). However, recent results of several multicenter large-scale randomized controlled trials showed that regional anesthesia (spinal, subarachnoid, or both techniques combined with no sedation) did not significantly reduce the risk of POD in geriatric patients with hip fractures compared to general anesthesia (Neuman et al., 2021; Li et al., 2022). These findings indicated that the decision of which anesthetic to use during hip fracture surgery may depend more on patient desire than on predicted variations in clinical results. Additionally, in a randomized controlled trial of 94 geriatric patients with hip fractures (Gu et al., 2021), ultrasound-guided multiple nerve block was shown to be a safe and more effective anesthetic technique than general anesthesia, with reduced intravenous opioid consumption and POD incidence. However, the POD incidence in this study was the secondary outcome, and it was assessed by the delirium index, which is not widely used in clinical practice.

When it comes to general anesthesia, there are contradictory views on the effects of intravenous and inhalation anesthesia on POD. Several studies suggested that inhalation anesthesia, such as sevoflurane anesthesia, may increase the risk of POD by inducing or exacerbating neuroinflammation (Zhang et al., 2013; Saller et al., 2022). On the contrary, a randomized controlled trial of 209 geriatric patients following total hip or knee replacements found that sevoflurane was superior to propofol in the reduction of POD duration (*p* = 0.049; Mei et al., 2020). However, a growing body of studies has revealed that neither the incidence nor the severity of POD changed significantly between intravenous and inhalation anesthesia (Royse et al., 2011; Lurati Buse et al., 2012; Miller et al., 2018; Jin et al., 2020). Future studies still need to further explore the effects of different anesthesia techniques on the incidence and severity of POD in geriatric patients with hip fractures.

### Pharmacological interventions

Pharmacologic strategies for the prevention and management of POD in geriatric patients with hip fractures have been studied for many years, while effective candidate drugs with strong evidence have not been found yet (Pluta et al., 2020). Drugs such as steroids (Swarbrick and Partridge, 2022), melatonin (Campbell et al., 2019), and vitamin D (Hung et al., 2022) have all been reported to be associated with POD, but all lack strong evidence. In the current, most studies focus on three medications: dexmedetomidine, antipsychotics, and analgesics.

#### Dexmedetomidine

It is reported that dexmedetomidine is an effective medication in the prevention of POD in hospitalized geriatric patients (León-Salas et al., 2020), but current studies mainly focus on the effect of dexmedetomidine on POD in geriatric patients after cardiovascular surgery. Dexmedetomidine was shown to be the most promising agent able to prevent the occurrence of POD in patients after cardiac surgeries, according to a 2020 meta-analysis of 56 RCTs that examined 38 interventions (Pieri et al., 2020). Two randomized controlled trials compared the effect of dexmedetomidine to midazolam and clonidine respectively, and both found that dexmedetomidine was more effective in the prevention of POD in patients who underwent stomatological or cardiovascular surgeries (Shokri and Ali, 2020; Wang et al., 2020). However, in a 2022 meta-analysis of 30 RCTs comprising 4,090 patients (Patel et al., 2022), perioperative dexmedetomidine administration failed to reduce the incidence of delirium in patients after cardiac surgeries. The possible explanation is that this meta-analysis included an increased number of trials, many of which have been published relatively recently and have not shown a beneficial relationship between dexmedetomidine and POD, and this study excluded trials at high risk of bias.

Whether dexmedetomidine is the most effective candidate for POD still needs further study. Additionally, adverse events such as bradycardia should be considered (Zeng et al., 2019). Future studies should focus on the effects of dexmedetomidine on POD in elderly patients with hip fractures to find effective and feasible dosing methods.

#### Analgesics

Acute pain in patients who underwent noncardiac surgery strongly mediates the relationship between preoperative cognitive impairment and postoperative delirium (Ma et al., 2022), therefore, adequate analgesia plays an essential role in the rehabilitation of geriatric hip fracture patients (Mears and Kates, 2015). Commonly used perioperative analgesic drugs include nonsteroidal anti-inflammatory drugs and opioids, narcotic and non-narcotic, respectively. Acetaminophen is an efficacious medication for analgesia in geriatric patients with hip fractures (Tsang et al., 2013; Bollinger et al., 2015). There is a retrospective study of 123 geriatric hip fracture patients (Connolly et al., 2020), which showed that intravenous acetaminophen had reduced the incidence of POD from 32.8 to 15.4% (*p* = 0.024). In a 2021 network meta-analysis (Lee et al., 2021), the combination of propofol and acetaminophen was the most successful pharmaceutical strategy with a minimal incidence of POD.

Although opioid administration can itself be a risk factor for POD (Duprey et al., 2021), pain is probably a stronger POD trigger in geriatric patients with hip fractures (Morrison et al., 2003). Two RCTs compared morphine and oxycodone to sufentanil respectively, and they found that both low-dose morphine intrathecal analgesia and oxycodone patient-controlled intravenous analgesia are superior to sufentanil patient-controlled intravenous analgesia in the prevention of POD in geriatric patients with hip fractures (Gan et al., 2020; Xu et al., 2022). Therefore, multimodal analgesia is recommended for the management of POD in geriatric patients with hip fractures.

#### Antipsychotics

Antipsychotics are dopamine D_2_ receptor antagonists, with varying degrees of affinity to muscarinic, serotonergic, and α_2_ adrenergic receptors (Farah, 2005). They can be divided into first-generation antipsychotics (FGAs) and second-generation antipsychotics (SGAs). FGAs are also known as typical antipsychotics and relate to increased risks of psychomotor complications; SGAs are also called atypical antipsychotics and are linked to higher chances of cardiovascular and metabolic complications (Jin et al., 2020). The effect of antipsychotics on POD in geriatric patients with hip fractures is still doubtful, owing to minimal evidence and variable complications of them (Pluta et al., 2020).

Haloperidol, a typical antipsychotic, was studied in multiple trials with inconsistent results (Igwe et al., 2020; Li et al., 2021; Tillemans et al., 2021). According to a meta-analysis of patients admitted to an ICU, haloperidol prophylaxis could lower the incidence of POD compared to the placebo (RR 0.63; *p* = 0.004; Lin et al., 2020). However, two massive prospective RCTs that compared haloperidol to ketamine or ziprasidone respectively, indicated that none of the three medications could shorten the frequency and duration of postoperative cognitive dysfunction and POD in patients with a critical illness (Girard et al., 2018; Hollinger et al., 2021). Some studies supported the efficacy of SGAs such as aripiprazole, risperidone, and olanzapine in the treatment of POD; nevertheless, the quality of the evidence is poor because of the heterogeneity and bias of the research (Tachi et al., 2021; Sugawara et al., 2022). Moreover, the safety of antipsychotics is more concerning than their efficacy. Boncyk et al. (2021) conducted a trial comparing haloperidol, olanzapine, and quetiapine in the management of delirious patients in the ICU. They reported that all three antipsychotics were associated with noticeably fewer days spent alive and out of the hospital, and similar results were also reported previously (Maust et al., 2015; Agar et al., 2017; Ralph and Espinet, 2018).

In conclusion, antipsychotics are mainly used in the management of POD in critically ill patients in the ICU, and future studies could focus on the effect of antipsychotics on POD in elderly patients with hip fractures.

#### Medication avoidance

Perioperative administration of certain medications has been considered an important risk factor for POD in geriatric patients with hip fractures, including antihistamines and scopolamine (Alagiakrishnan and Wiens, 2004; By the 2019 American Geriatrics Society Beers Criteria® Update Expert Panel, 2019). The most investigated perioperative medications with POD risk are benzodiazepines, antidepressants, and gabapentinoids.

Benzodiazepine receptor agonists (BZDRAs) are commonly used sedatives during the perioperative period, which can lower the consumption of intraoperative anesthetics (Nakagawa et al., 2000). However, numerous studies have demonstrated that perioperative usage of BZDRAs is linked to a higher risk of POD in geriatric patients with hip fractures (Poeran et al., 2020; Hernandez et al., 2021; Duprey et al., 2022; Lertkovit et al., 2022). The concrete mechanisms of benzodiazepine-induced POD vary based on the subtype of BZDRAs: Diazepam may affect the chloride plasticity mediated by Na^+^ − K^+^ − 2Cl-cotransporter isoform 1 and result in the gamma-aminobutyric acid α receptor malfunction (Matsumoto et al., 2021); midazolam can alter the expression and activity of acetyl-and butyryl-cholinesterase genes (Rump et al., 2022), and both contribute to benzodiazepine - induced POD. Additionally, a retrospective cohort study of 250 patients who were scheduled for surgery under general anesthesia investigated the relationship between the time course of BZDRAs use and the incidence of POD. It was discovered that the prevalence of POD was highest in the patients who stopped taking BZDRAs after surgery, which indicated that abrupt discontinuation of BZDRAs should be avoided during the perioperative period (Omichi et al., 2021).

Antidepressant administration during the perioperative period is also associated with a high incidence of POD in geriatric patients with hip fractures. In a matched case–control study of geriatric patients after hip or knee surgeries (Kassie et al., 2022), exposures to selective serotonin reuptake inhibitors (SSRIs) such as sertraline, citalopram, escitalopram, and fluvoxamine before surgery in cases were considerably greater than those in controls, which indicated that reduced preoperative use of SSRIs may prevent the occurrence of POD. Additionally, gabapentin and gabapentin combined with midazolam have also been found to be associated with a considerably increased risk of POD, according to several massive observatory studies in patients following major noncardiac surgeries (Athanassoglou et al., 2022; Duprey et al., 2022; Park et al., 2022). However, whether gabapentin can increase the incidence of POD in geriatric patients with hip fractures still needs further study.

## Conclusion

Postoperative delirium in geriatric patients with hip fractures is significantly related to high short - and long-term mortality as well as poor functional and cognitive recovery. The concrete mechanism of POD is still elusive, and early identification of patients with POD risks is imperative. In the current, prevention interventions such as risk prediction and orthogeriatric comprehensive care are the most effective measures for POD management. Dexmedetomidine prophylaxis and multimode analgesia may be effective for reducing the prevalence and severity of POD in geriatric patients with hip fractures, which needs more large-scale multicentral RCTs to investigate their safety and efficacy.

## Author contributions

YC and JL conceived the idea of this review. YC drafted the manuscript and created the figures. SL, HW, SD, FW, and CL performed the literature search and reviewed the content of this manuscript. All authors read and approved the final manuscript.

## Funding

This study was financially supported by the National first-class undergraduate professional construction project (clinical medicine) sub-project of Anhui Medical University, Natural Science Foundation of Hefei City (grant number: 2022041 to JL), Clinical Research cultivation Program of the Second Affiliated Hospital of Anhui Medical University (grant number: 2020LCZD20 to JL), and Basic and Clinical Cooperative Research Promotion Plan of Anhui Medical University (grant number: 2020xkjT040 to JL).

## Conflict of interest

The authors declare that the research was conducted in the absence of any commercial or financial relationships that could be construed as a potential conflict of interest.

## Publisher’s note

All claims expressed in this article are solely those of the authors and do not necessarily represent those of their affiliated organizations, or those of the publisher, the editors and the reviewers. Any product that may be evaluated in this article, or claim that may be made by its manufacturer, is not guaranteed or endorsed by the publisher.
